# Association between Low-Grade Albuminuria and Cardiovascular Risk in Korean Adults: The 2011–2012 Korea National Health and Nutrition Examination Survey

**DOI:** 10.1371/journal.pone.0118866

**Published:** 2015-03-05

**Authors:** Jae Won Hong, Cheol Ryong Ku, Jung Hyun Noh, Kyung Soo Ko, Byoung Doo Rhee, Dong-Jun Kim

**Affiliations:** 1 Department of Internal Medicine, Ilsan-Paik Hospital, College of Medicine, Inje University, Koyang, Gyeonggi-do, Republic of Korea; 2 Endocrinology, Yonsei University College of Medicine, Seoul, South Korea; 3 Department of Internal Medicine, Sanggye Paik Hospital, Cardiovascular and Metabolic Disease Center, College of Medicine, Inje University, Seoul, Republic of Korea; School of Public Health of University of São Paulo, BRAZIL

## Abstract

**Background:**

Recent studies have indicated that low UACR levels (<30 μg/mg) previously considered to be in the normal range (‘low-grade albuminuria’) are associated with cardiovascular morbidity and mortality in the general population.

**Methods:**

We studied 9,736 participants with albuminuria in the normal range from the 2011–2012 Korea National Health and Nutrition Examination Survey (KNHANES).

**Results:**

The weighted prevalences of metabolic syndrome (MS) and the 10-year risk for coronary heart disease measured using the Framingham risk score (FRS) ≥ 20% (high risk) were 22.5 ± 0.7% and 14.5 ± 0.7%, respectively, in males and 23.3 ± 0.8% and 8.5 ± 0.4%, respectively in females. Weighted comparisons among the tertiles of UACR revealed that the prevalences of MS and high-risk FRS increased with increasing UACR (MS: males, 15.9 ± 1.1, 20.2 ± 1.2, 32.4 ± 1.5%, respectively; *P * < 0.001; and females, 17.6 ± 1.0, 22.7 ± 1.0, 30.2 ± 1.4%, respectively; *P* < 0.001. High-risk FRS: males, 9.5 ± 0.7, 12.3 ± 0.9, 22.5 ± 1.2, respectively; *P* < 0.001; and females, 5.8 ± 0.6, 7.9 ± 0.7, 12.0 ± 0.9%, respectively; *P* < 0.001). The positive association persisted after adjusting for hypertension and diabetes. The weighted comparisons among the deciles of UACR revealed that the prevalences of MS and high-risk FRS began to increase at the ranges of 3.89–5.15 and 5.16–7.36 mg/g Cr, respectively.

**Conclusion:**

Low-grade albuminuria was significantly associated with estimated cardiovascular risk and MS in a nationally representative sample of Koreans.

## Introduction

Microalbuminuria is defined as a urinary albumin-to-creatinine ratio (UACR) between 30 and 300 μg/mg in spot samples. Microalbuminuria is a well-known risk factor for overt proteinuria and end-stage renal disease in subjects with hypertension or diabetes and is associated with cardiovascular morbidity and mortality in individuals with or without diabetes [1–3].

However, recent findings have indicated that low UACR levels (<30 μg/mg) previously considered to be in the normal range (‘low-grade albuminuria’) are associated with cardiovascular morbidity and mortality in the general population [4–7]. Hillege *et al*. [4] found a positive dose-response relationship between increasing urinary albumin excretion and all-cause mortality in their Prevention of Renal and Vascular End Stage Disease (PREVEND) cohort. The relationship was apparent at levels of albuminuria currently considered normal. The Danish World Health Organization Multinational “Monitoring of trends and determinants in cardiovascular disease” (WHO MONICA) project found that a slight increase in the UACR (>5.8 mg/g) was a clinically relevant marker of ischemic heart disease risk after adjusting for established atherosclerotic risk factors such as male sex, hypertension, dyslipidemia, smoking, old age, and obesity [5]. In The Framingham Heart Study, low-grade urinary albumin excretion was associated with increased risk of cardiovascular disease and mortality in nonhypertensive, nondiabetic individuals [6].

However, few studies have investigated the association of low-grade albuminuria with cardiovascular risk factors and/or related components in the general Asian population [8–11].

Thus, we estimated cardiovascular risk using the Framingham Risk Score (FRS) and investigated the association between low-grade albuminuria and estimated cardiovascular risk using data from the Korea National Health and Nutrition Examination Survey (KNHANES) for 2011–2012.

## Methods

### Study population and data collection

Our study used data from the 2011–2012 KNHANES, a cross-sectional and nationally representative survey conducted by the Korean Center for Disease Control for Health Statistics. The KNHANES has been conducted periodically since 1998 to assess the health and nutritional status of the civilian non-institutionalized Korean population. Participants were chosen using proportional allocation-systemic sampling with multistage stratification. A standardized interview was conducted in the homes of the participants to collect information on demographic variables, family history, medical history, medications used, and a variety of other health-related variables. The health interview included well-established questions to determine the demographic and socioeconomic characteristics of the subjects including questions on age, education level, occupation, income, marital status, smoking habit, alcohol consumption, exercise, previous and current diseases, and family disease history.

Subjects were questioned about whether they exercised with an intensity that left them sweating and with slight difficulty breathing. Subjects who exercised regularly at a moderate intensity were asked about the frequency at which they exercised per week and the length of time per exercise session. Regular exercise was defined as exercising five or more times per week. Alcohol consumption was assessed by questioning the subjects about their drinking behavior during the month before the interview. Heavy alcohol drinking was categorized as drinking four or more times per week. Hypertension was defined as systolic blood pressure ≥ 140 mmHg, diastolic blood pressure ≥ 90 mmHg, or use of antihypertensive medications irrespective of blood pressure. Diabetes was defined as fasting plasma glucose (FPG) ≥ 7.0 mmol/L, current use of anti-diabetes medication, or a previous diagnosis of diabetes by a doctor. Obesity was defined as a body mass index (BMI) ≥ 25 kg/m^2^ according to the Asia-Pacific obesity classification guidelines [12].

Height and weight were obtained using standardized techniques and equipment. Height was measured to the nearest 0.1 cm using a portable stadiometer (Seriter, Bismarck, ND, USA). Weight was measured to the nearest 0.1 kg using a Giant-150N calibrated balance-beam scale (Hana, Seoul, Korea). BMI was calculated by dividing weight by the square of height (kg/m^2^). Systolic and diastolic blood pressures were measured using a standard sphygmomanometer with the patient in the sitting position. Three measurements were made for all subjects at 5-min intervals; the average of the second and third measurements was used in the analysis.

### Laboratory analysis

Blood samples were collected in the morning after fasting for at least 8 h. FPG, total cholesterol, triglycerides (TGs), low-density lipoprotein cholesterol (LDL-C), high-density lipoprotein cholesterol (HDL-C), and serum creatinine levels were measured using a Hitachi Automatic Analyzer 7600 (Hitachi, Tokyo, Japan). Urine albumin and creatinine concentrations were measured in the same laboratory for all surveys. Serum and urinary concentrations of creatinine were measured using a colorimetric method (Hitachi Automatic Analyzer 7600, Hitachi). The inter-assay coefficient of variation for serum creatinine was <1.4%. Urinary albumin was measured in random urine samples using a turbidimetric immunoassay (Hitachi Automatic Analyzer 7600, Hitachi). Laboratory control measures used in the KNHANES indicated that these assays were highly reliable with consistently low coefficients of variation (3.1%). The UACR was reported as the albumin-creatinine ratio in milligrams per gram of creatinine (mg/g Cr). The cut-off level for albuminuria was ≥30 mg/g Cr (microalbuminuria was defined as UACR = 30–299 mg/g Cr, and macroalbuminuria was defined as UACR ≥300 mg/g Cr) [13]. The estimated glomerular filtration rate (eGFR) was calculated using the abbreviated equation from the Modification of Diet in Renal Disease (MDRD) study: eGFR (mL/min/1.73 m^2^) = 175 × (S_Cr_/88.4, μmol/L)^-1.154^ × Age^-0.203^ × 0.742 (if female) [14]. Glycated hemoglobin (HbA1c) was measured using high-performance liquid chromatography (HLC-723G7, Tosoh, Tokyo, Japan). The detailed methods for comparing and verifying the validity and reliability for each survey are described elsewhere [15].

### Definitions

According to the Adult Treatment Panel (ATP) III criteria using waist circumference cutoff modifications for Asian populations as suggested by the Asia-Pacific guidelines [16], metabolic syndrome was defined as having three or more of the following factors: (1) Central obesity: waist circumference ≥ 90 cm in men and ≥80 cm in women; (2) Hypertriglycemia: TG ≥ 150mg/dL; (3) HDL-C <40mg/dL in men and <50 mg/dL in women; (4) Hypertension: Blood pressure ≥ 130/85mmHg or taking antihypertensive medication; (5) Hyperglycemia: FPG ≥ 100 mg/dL or taking antidiabetic medication.

The FRS is the gold standard for predicting an individual’s risk for developing cardiovascular disease over the next 10 years [17]. In 2002, the ATP III modified the FRS scoring system to include age, sex, total cholesterol, HDL-C, systolic blood pressure, and smoking status [18].

Based on the FRS, we categorized the 10-year risk for coronary heart disease (CHD) as high risk (>20%), intermediate risk (10–20%), and low risk (<10%). An FRS ≥ 20% is considered to be a CHD risk equivalent, and primary prevention is recommended for individuals in this category [18].

The sex-specific tertile cutoff points for the UACR were: first tertile, 0–1.61 (males) and 0–2.12 mg/g (females); second tertile, 1.62–4.01 (males) and 2.13–5.26 (females) mg/g; and third tertile, 4.02–29.9 (males) and 5.27–29.9 mg/g (females). The decile ranges of UACR were: first, 0–0.27 mg/g; second, 0.28–1.01 mg/g; third, 1.02–1.61 mg/g; fourth, 1.62–2.24 mg/g; fifth, 2.25–2.95 mg/g; sixth, 2.96–3.88 mg/g; seventh, 3.89–5.15 mg/g; eighth, 5.16–7.36 mg/g; ninth, 7.37–12.09 mg/g; and tenth, 12.10–29.99 mg/g.

### Ethics statement

The present study was approved by the Institutional Review Board of Ilsan Paik Hospital, Republic of Korea (IB-2-1406-026). After approval of the study proposal, the KNHANES dataset was made available at the request of the investigator. Because the dataset did not include any personal information and the participants’ consent had already been given for the KNHANES, our study was exempt from participant consent.

### Statistical analyses

The KNHANES participants were not randomly sampled. The survey was designed using a complex, stratified, multistage probability-sampling model; thus, individual participants were not equally representative of the Korean population. To obtain representative prevalence rates from the dataset, it was necessary to consider the power of each participant (sample weight) as representative of the Korean population. Following approval from the Korea Centers for Disease Control and Prevention, we received a survey dataset that included information about the survey location, strata by age, sex, and various other factors, and the sample weight for each participant. The survey sample weights, calculated taking into account the sampling rate, response rate, and age/sex proportions of the reference population (2005 Korean National Census Registry), were used in all analyses to provide representative estimates of the non-institutionalized Korean civilian population. Statistical tests were performed using the Statistical Package for the Social Sciences software, ver. 21.0 for Windows (SPSS Inc., Chicago, IL, USA).

**Fig 1:**
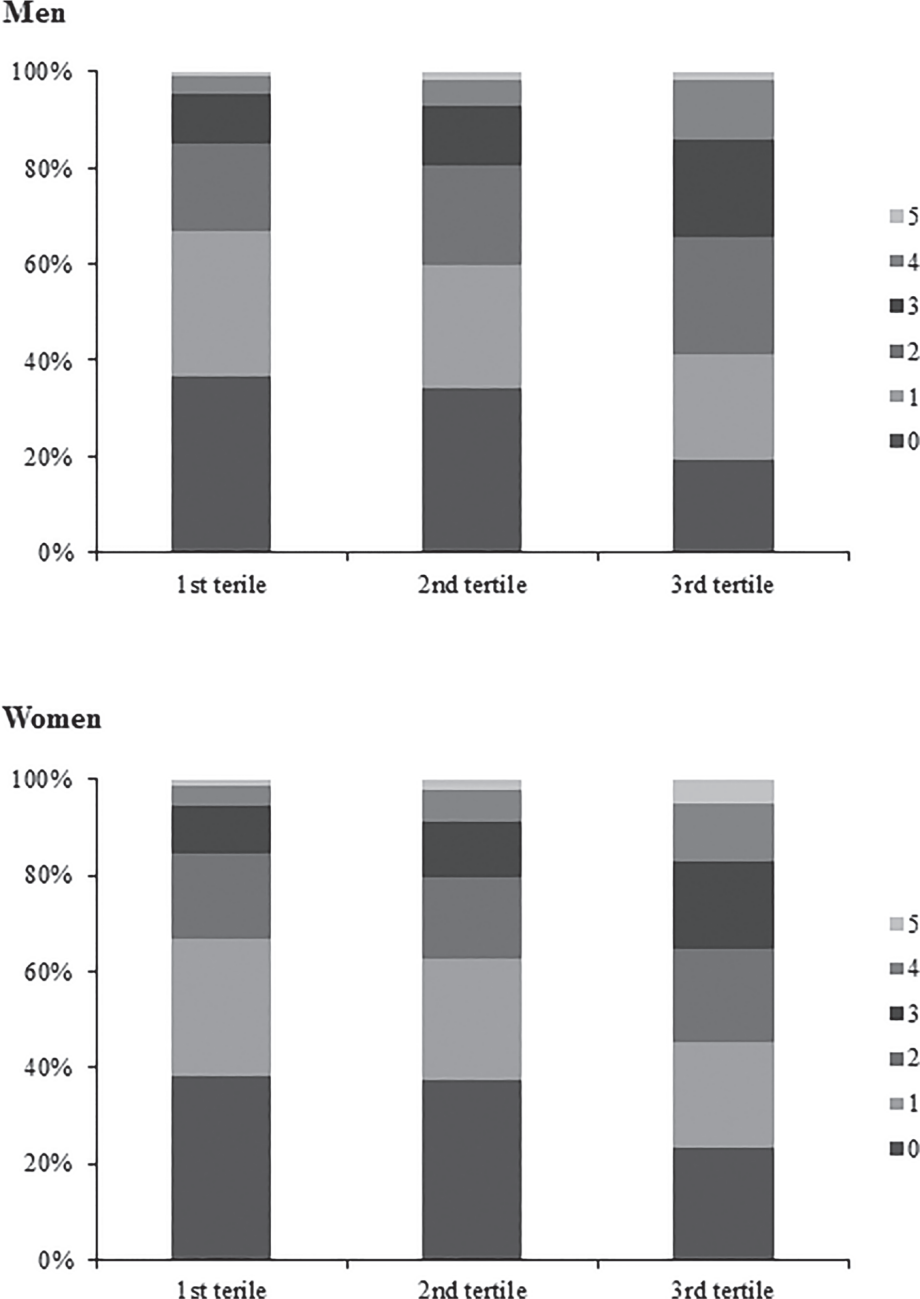
Number of components in metabolic syndrome by tertile of albuminuria The number of metabolic syndrome components increased according to tertile of Urinary albumin-creatinine ratio (UACR) (*P* < 0.001) in men and women.

**Fig 2:**
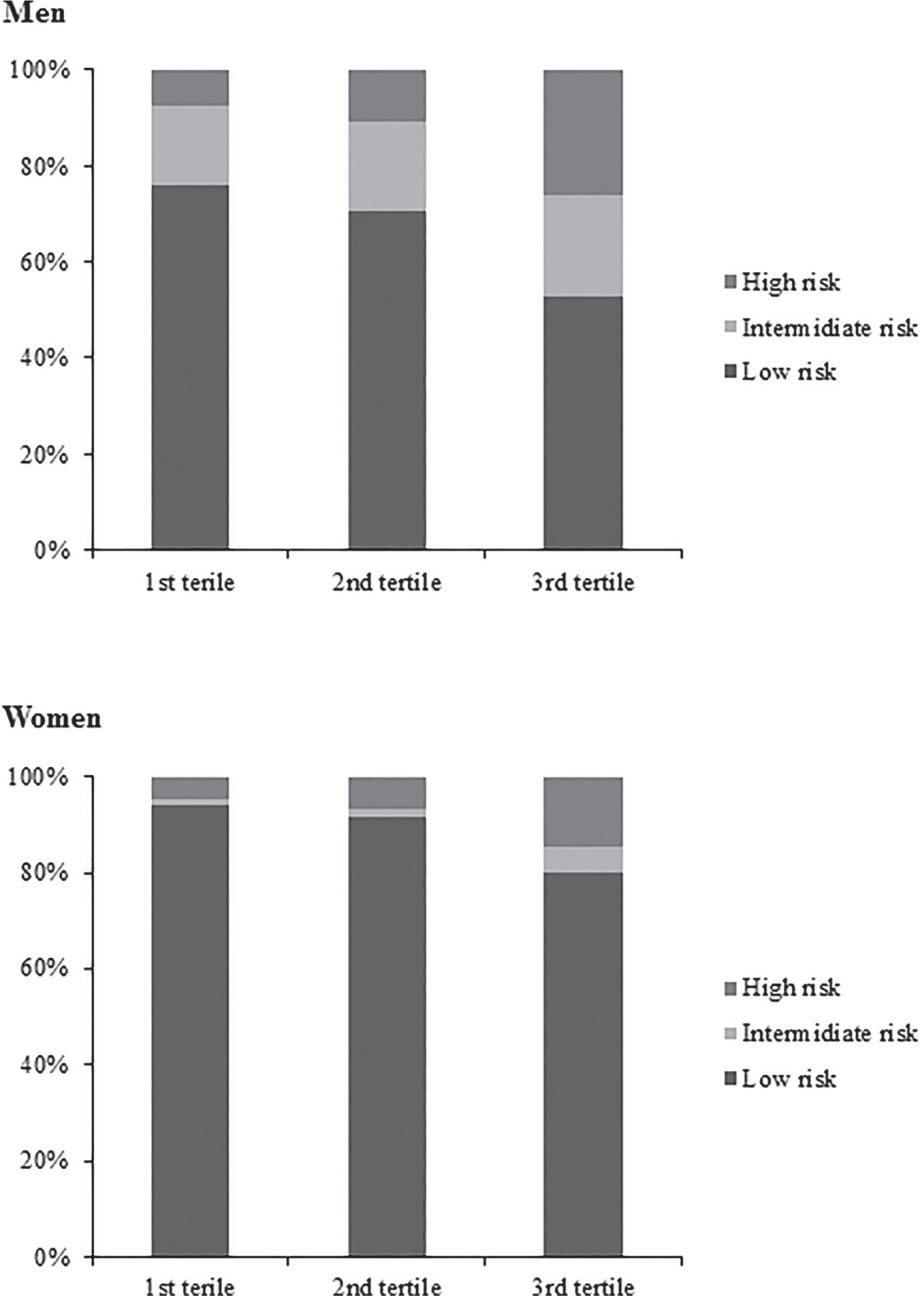
Weighted and unadjusted distribution of Framingham 10-year risk by tertile of albuminuria. 10-year risk for coronary heart disease according to the Framingham score >20%; high risk, 10–20%; intermediate risk and <10%; low risk.

**Fig 3:**
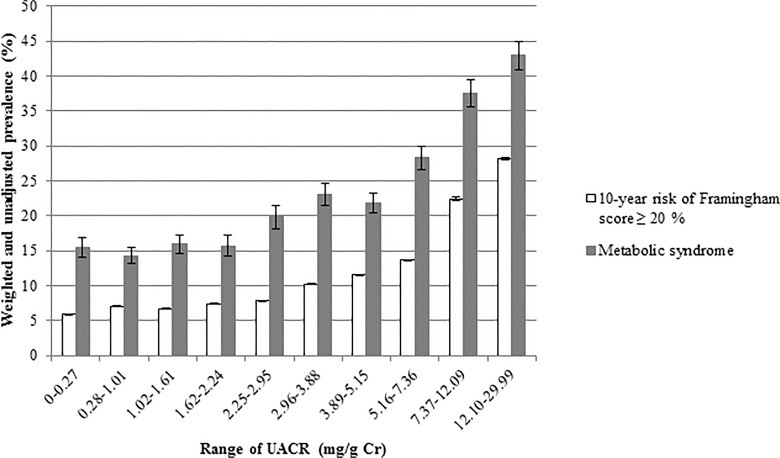
Weighted and unadjusted the prevalence of metabolic syndrome and 10-year risk of Framingham score ≥ 20% (high-risk FRS) by deciles of albuminuria. The weighted comparisons among the deciles of Urinary albumin-creatinine ratio (UACR) revealed that the prevalences of metabolic syndrome and high-risk FRS began to increase at the ranges of 3.89–5.15 (*P* = 0.004) and 5.16–7.36 mg/g Cr (*P* < 0.001), respectively.

To compare weighted age between men and women, Student’s *t* test was performed (Table 1). We compared age-adjusted weighted demographic and clinical characteristics between men and women using an analysis of covariance (ANCOVA) (Table 1).To compare age-adjusted weighted demographic and clinical characteristics among groups according to tertiles of the UACR, ANCOVA was performed in each sex (Tables 2 and 3). A logistic regression analysis was used to evaluate the odds ratios for highest tertile of UACR as covariates with sex, current smoking, obesity, hypertension, diabetes, hypertriglycemia (Table 4). Comparison of weighted prevalence of 10-year risk of Framingham score ≥ 20% according to tertile of the UACR was analyzed using an ANCOVA (Table 5). Comparison of the prevalence and number of metabolic syndrome components and distribution of the Framingham 10-year CHD risk (low, intermediate, and high risk) by tertile (Figs. 1 and 2) and decile (Fig. 3) of the UACR were analyzed using the chi-square test. All tests were two-sided, and *P*-values < 0.05 were considered to indicate statistical significance.

**Table 1:** Weighted age, sex, and age-adjusted demographic and clinical characteristics of the Korean population ≥19 years old within normal range of albuminuria* in the 2011–2012 KNHANES.

	Total	Men	Women	*P* for men vs. women
*N*, (unweighted/weighted)	9736/31733439	4376/16876139	5360/14857300	
Age (years)	45.0 ± 0.3	46.5 ± 0.3	46.5 ± 0.3	<0.001
Men (%)	53.2 ± 0.5			
Current smoking (%)	26.9 ± 0.6	44.2 ± 0.9	7.2 ± 0.5	<0.001
Heavy alcohol drinking (%)	7.3 ± 0.4	11.9 ± 0.6	2.1 ± 0.3	<0.001
BMI (kg/m^2^)	23.7 ± 0.1	24.1 ± 0.1	23.3 ± 0.1	<0.001
Obesity (%)	31.9 ± 0.7	35.1 ± 0.9	28.3 ± 0.9	<0.001
Systolic BP (mmHg)	117.1 ± 0.2	119.3 ± 0.3	114.6 ± 0.3	<0.001
Diastolic BP (mmHg)	76.1 ± 0.2	78.7 ± 0.2	73.1 ± 0.2	<0.001
Anti-hypertensive drug (%)	12.8 ± 0.4	11.2 ± 0.5	14.7 ± 0.6	<0.001
Hypertension (%)	24.2 ± 0.6	26.2 ± 0.8	21.6 ± 0.8	<0.001
FPG (mg/dl)	96.0 ± 0.3	97.4 ± 0.3	94.5 ± 0.4	<0.001
Diabetes (%)	8.7 ± 0.3	9.3 ± 0.5	8.1 ± 0.4	0.067
Serum Total cholesterol (mg/dl)	189.1 ± 0.5	187.8 ± 0.8	190.1 ± 0.7	0.004
Serum triglyceride (mg/dl)	133.6 ± 1.6	151.6 ± 2.3	113.1 ± 1.9	<0.001
Serum HDL-cholesterol (mg/dl)	52.4 ± 0.2	49.4 ± 0.2	56.7 ± 0.2	<0.001
eGFR, ml/min/1.73m^2^	97.0 ± 0.3	95.3 ± 0.3	98.9 ± 0.3	<0.001
UACR (mg/g Cr)	2.96 (1.33–6.08)	2.54 (1.19–5.17)	3.39 (1.48–6.70)	<0.001
Metabolic syndrome (%)	22.9 ± 0.5	22.5 ± 0.7	23.3 ± 0.8	0.433
Framingham risk score (point)	6.7 ± 0.1	6.5 ± 0.2	6.8 ± 0.2	0.078
10-year CHD risk ≥ 20% (%)	11.7 ± 0.4	14.5 ± 0.7	8.5 ± 0.4	<0.001

* Urinary albumin-creatinine ratio (UACR) < 30 mg/g Cr.

Data are expressed as means with SEM, except for UACR expressed as median with interquartile range. BMI, body mass index; BP, blood pressure; FPG, fasting plasma glucose; HDL, high-density lipoprotein; eGFR, estimated glomerular filtration rate; CHD, coronary heart disease.

**Table 2:** Weighted age and age-adjusted demographic and clinical characteristics of Korean adult men within normal range of albuminuria* by tertile of albuminuria.

	1^st^ tertile	2^nd^ tertile	3^rd^ tertile	*P* for difference
Range of UACR (mg/g Cr)	0–1.61	1.62–4.01	4.02–29.9	
*N*, (unweighted/weighted)	1,461/5,646,232	1,464/6,061,047	1,451/5,168,860	
Age (years)	41.4 ± 0.5	41.9 ± 0.5	48.1 ± 0.6	<0.001
Heavy alcohol drinking (%)	10.6 ± 0.9	12.2 ± 1.1	13.0 ± 1.1	0.266
Regular exercise (%)	10.3 ± 1.1	9.7 ± 1.0	9.6 ± 1.0	0.898
Current smoking (%)	40.5 ± 1.7	43.9 ± 1.6	48.5 ± 1.7	0.002
Waist circumference (cm)	83.2 ± 0.3	83.5 ± 0.3	85.4 ± 0.3	<0.001
BMI (kg/m^2^)	23.8 ± 0.1	24.0 ± 0.1	24.6 ± 0.1	<0.001
Obesity (%)	31.3 ± 1.6	33.6 ± 1.6	41.0 ± 1.6	<0.001
Systolic BP (mmHg)	117.4 ± 0.4	118.1 ± 0.4	122.8 ± 0.6	<0.001
Diastolic BP (mmHg)	77.5 ± 0.3	78.0 ± 0.3	80.7 ± 0.5	<0.001
Anti-hypertensive drugs (%)	9.1 ± 0.6	10.3 ± 0.7	14.7 ± 1.0	<0.001
Hypertension (%)	21.3 ± 1.1	22.4 ± 1.3	35.9 ± 1.7	<0.001
FPG (mg/dl)	94.3 ± 0.4	95.4 ± 0.4	103.0 ± 0.8	<0.001
HbA1c (%)	5.55 ± 0.01	5.62 ± 0.02	5.83 ± 0.03	<0.001
Anti-diabetes drugs (%)	2.4 ± 0.4	3.5 ± 0.5	7.7 ± 0.8	<0.001
Diabetes (%)	5.1 ± 0.6	7.0 ± 0.7	16.6 ± 1.0	<0.001
Serum total cholesterol (mg/dl)	186.0 ± 1.1	188.8 ± 1.1	188.5 ± 1.6	0.154
Serum Triglyceride (mg/dl)	139.6 ± 3.9	143.3 ± 3.2	174.3 ± 5.1	<0.001
Serum HDL-cholesterol (mg/dl)	50.4 ± 0.4	49.1 ± 0.4	48.7 ± 0.4	0.010
Anti-lipid drugs (%)	2.9 ± 0.4	3.1 ± 0.5	4.0 ± 0.6	0.267
AST (U/l)	23.3 ± 9.7	23.6 ± 0.4	26.2 ± 0.6	<0.001
ALT (U/l)	24.6 ± 1.2	25.8 ± 1.0	30.9 ± 1.1	<0.001
WBC (/mm^3^)	6164 ± 50	6359 ± 54	6621 ± 59	<0.001
eGFR (ml/min/1.73m^2^)	94.2 ± 0.4	95.6 ± 0.4	96.1 ± 0.5	0.005
Metabolic syndrome (%)	15.9 ± 1.1	20.2 ± 1.2	32.4 ± 1.5	<0.001
Framingham risk score (point)	6.1 ± 0.1	6.4 ± 0.2	7.0 ± 0.2	<0.001
10-year CHD risk ≥ 20% (%)	9.5 ± 0.7	12.3 ± 0.9	22.5 ± 1.2	<0.001

* Urinary albumin-creatinine ratio (UACR) < 30 mg/g Cr.

Data are expressed as means with SEM. BMI, body mass index; BP, blood pressure; FPG, fasting plasma glucose; HDL, high-density lipoprotein; eGFR, estimated glomerular filtration rate; CHD, coronary heart disease.

**Table 3:** Weighted age and age-adjusted demographic and clinical characteristics of Korean adult women within normal range of albuminuria* by tertile of albuminuria.

	1^st^ tertile	2^nd^ tertile	3^rd^ tertile	*P* for difference
Range of UACR (mg/g Cr)	0–2.12	2.13–5.26	5.27–29.9	
*N*, (unweighted/weighted)	1,795/4,958,729	1,788/5,209,584	1,777/4,688,987	
Age (years)	44.3 ± 0.4	44.0 ± 0.4	51.5 ± 0.6	<0.001
Heavy alcohol drinking (%)	2.1 ± 0.5	2.0 ± 0.4	2.2 ± 0.5	0.929
Regular exercise (%)	8.3 ± 0.8	7.3 ± 0.8	5.7 ± 0.8	0.045
Current smoking (%)	6.5 ± 0.8	7.4 ± 0.8	7.7 ± 0.8	0.560
Waist circumference (cm)	78.0 ± 0.3	77.8 ± 0.3	78.4 ± 0.4	0.293
BMI (kg/m^2^)	23.3 ± 0.1	23.3 ± 0.1	23.5 ± 0.1	0.420
Obesity (%)	24.5 ± 1.3	28.1 ± 1.2	31.5 ± 1.5	0.005
Systolic BP (mmHg)	111.8 ± 0.4	113.9 ± 0.4	118.4 ± 0.5	<0.001
Diastolic BP (mmHg)	71.8 ± 0.3	72.9 ± 0.3	74.6 ± 0.3	<0.001
Anti-hypertensive drugs (%)	10.8 ± 0.7	14.3 ± 0.8	19.2 ± 1.0	<0.001
Hypertension (%)	15.4 ± 0.9	20.0 ± 0.9	30.1 ± 1.1	<0.001
FPG (mg/dl)	92.6 ± 0.4	93.8 ± 0.5	97.3 ± 0.8	<0.001
HbA1c (%)	5.56 ± 0.01	5.63 ± 0.02	5.75 ± 0.03	<0.001
Anti-diabetes drugs (%)	3.1 ± 0.5	3.6 ± 0.5	5.9 ± 0.7	0.005
Diabetes (%)	5.6 ± 0.6	7.6 ± 0.7	11.5 ± 0.9	<0.001
Serum total cholesterol (mg/dl)	190.3 ± 1.0	191.6 ± 1.0	190.0 ± 1.2	0.504
Serum Triglyceride (mg/dl)	109.2 ± 1.8	112.0 ± 2.2	118.4 ± 4.8	0.174
Serum HDL-cholesterol (mg/dl)	56.2 ± 0.4	55.7 ± 0.4	55.2 ± 0.4	0.211
Anti-lipid drugs (%)	4.3 ± 0.5	5.5 ± 0.5	5.9 ± 0.7	0.073
AST (U/l)	19.8 ± 0.3	19.8 ± 0.2	21.1 ± 0.4	0.014
ALT (U/l)	17.1 ± 0.4	17.0 ± 0.4	19.0 ± 0.6	0.012
WBC (/mm^3^)	5658 ± 42	5755 ± 50	5822 ± 57	0.052
eGFR (ml/min/1.73m^2^)	97.8 ± 0.4	99.0 ± 0.4	100.0 ± 0.4	<0.001
Metabolic syndrome (%)	17.6 ± 1.0	22.7 ± 1.0	30.2 ± 1.4	<0.001
Framingham risk score (point)	6.5 ± 0.1	6.9 ± 0.1	7.1 ± 0.1	0.003
10-year CHD risk ≥ 20% (%)	5.8 ± 0.6	7.9 ± 0.7	12.0 ± 0.9	<0.001

* Urinary albumin-creatinine ratio (UACR) < 30 mg/g Cr.

Data are expressed as means with SEM. BMI, body mass index; BP, blood pressure; FPG, fasting plasma glucose; HDL, high-density lipoprotein; eGFR, estimated glomerular filtration rate; CHD, coronary heart disease.

**Table 4:** Logistic regression analysis for the highest tertile of UACR.

		Odd ratio (95% CI)	*P*
Age	19–39 years	reference	
	40–64 years	1.23 (1.07–1.40)	0.004
	65-years	2.13 (1.81–2.51)	<0.001
Men		0.50 (0.45–0.57)	<0.001
Current smoking		1.25 (1.07–1.47)	0.005
Obesity		1.08 (0.94–1.24)	0.275
Hypertension		2.00 (1.76–2.28)	<0.001
Diabetes		2.37 (1.93–2.90)	<0.001
Serum triglyceride ≥ 150 mg/dl		1.23 (1.08–1.40)	0.002

Urinary albumin-creatinine ratio (UACR)

**Table 5:** Weighted prevalence of 10-year risk of Framingham score ≥ 20% by tertile of albuminuria.

	Tertile 1	Tertile 2	Tertile 3	*P*
Range of UACR (mg/g Cr)	0–0.34	0.35–4.64	4.65–29.99	
No adjusted	6.7 ± 0.1	8.6 ± 0.1	20.2 ± 0.1	<0.001
Age, sex-adjusted				
no exclusion	7.2 ± 0.1	10.0 ± 0.1	17.7 ± 0.1	<0.001
exclusion of anti-hypertensive medication	4.7 ± 0.1	6.9 ± 0.1	13.4 ± 0.1	<0.001
exclusion of hypertension	4.0 ± 0.1	5.9 ± 0.1	12.4 ± 0.1	<0.001
exclusion of diabetes	2.2 ± 0.1	3.1 ± 0.1	4.3 ± 0.1	0.001
exclusion of eGFR < 60 (ml/min/1.73m^2^)	6.9 ± 0.1	9.4 ± 0.1	17.1 ± 0.1	<0.001

Urinary albumin-creatinine ratio (UACR)

## Results

### Demographic and clinical characteristics of the study population

The weighted demographic and clinical characteristics of the study population are shown in Table 1.

Of the 11,473 adults (≥19 years of age) who underwent the health interview and laboratory examination for the 2011–2012 KNHANES, 1,737 had micro- or macroalbuminuria and were excluded. Our study included 9,736 subjects with albuminuria in the normal range.

The weighted age of participants was 45.0 ± 0.3 (mean ± SEM) years and the weighted percentage of men was 53.2 ± 0. 5 (mean ± SEM). The weighted mean UACR and FRS were 2.96 mg/g Cr and 6.7 ± 0.1 point, respectively. The weighted prevalences of metabolic syndrome and 10-year CHD risk according to an FRS ≥ 20% (high-risk FRS) were 22.5 ± 0.7% and 14.5 ± 0.7% in males and 23.3 ± 0.8% and 8.5 ± 0.4% in females, respectively.

The sex-related comparisons revealed that the percentage of current smoking, heavy alcohol drinking, obesity, and hypertension was higher in males than in females (*P* < 0.001). BMI and serum TG levels were higher in males than in females. Serum total cholesterol and HDL-C were higher in females than in males. The average UACR in males was 2.54 mg/g Cr, which was significantly lower than that of females (3.39 mg/g Cr; *P* < 0.001). The percentage of males in the high-risk FRS category was higher than that of females (14.5 ± 0.7% vs. 8.5 ± 0.4%, respectively; *P* < 0.001).

### Demographic and clinical characteristics according to tertile of low-grade albuminuria

The weighted age and age-adjusted demographic and clinical characteristics of Korean adult males within the normal range of albuminuria are shown in Table 2 according to tertile of UACR. A total of 4,376 males were divided into three categories according to tertile of UACR. FPG, HbA1c, and white blood cell (WBC) levels and the percentages of diabetes, metabolic syndrome, and high-risk FRS increased as albuminuria increased (*P* < 0.05). Waist circumference, BMI, and the percentages of obesity, hypertension, liver enzyme, serum TG, and FRS were highest in third tertile UACR (*P* < 0.01); however, values did not differ between the first and second tertiles of UACR.

In females (unweighted *n* = 5360/weighted *n* = 14857300), the percentage of hypertension, diabetes, metabolic syndrome, and high-risk FRS increased according to UACR tertiles, which was similar to the results for males (Table 3). Furthermore, HbA1c and eGFR levels were significantly positively correlated with increasing tertiles of UACR (*P*< 0.001). FPG and liver enzyme levels were highest in the third tertile UACR (*P* = 0.006); however, we found no difference in the values between the first and second tertiles of UACR. The FRS was lowest in first tertile of UACR (*P* = 0.046); however, it was not significantly different between the second and third tertiles (*P* = 0.08).

The logistic regression analysis revealed that age, female sex, current smoking, hypertension, diabetes, and serum TG ≥ 150 mg/dL were associated with the highest tertile of UACR (Table 4).

### Metabolic syndrome and 10-year CHD risk according to tertile of low-grade albuminuria

The weighted and unadjusted prevalence of metabolic syndrome increased according to tertile of UACR in both sexes (males: 15.9 ± 1.1, 20.2 ± 1.2, and 32.4 ± 1.5%, respectively; *P* < 0.001; females: 17.6 ± 1.0, 22.7 ± 1.0, and 30.2 ± 1.4%, respectively; *P* < 0.001). The number of metabolic syndrome components increased according to tertile of UACR (*P* < 0.001; Fig. 1). The weighted and unadjusted distribution of the 10-year CHD risk according to FRS is shown in Fig. 2.

The age- and sex-adjusted weighted percentage of high-risk FRS increased as the tertile of UACR increased (7.2 ± 0.1%, 10.0 ± 0.1%, and 17.7 ± 0.1%, respectively; *P* < 0.001). This trend persisted after adjusting for anti-hypertensive medication use (4.7 ± 0.1%, 6.9 ± 0.1%, and 13.4 ± 0.1%, respectively; *P* < 0.001), hypertension (4.0 ± 0.1%, 5.9 ± 0.1%, and 12.4 ± 0.1%, respectively; *P* < 0.001), and diabetes (2.2 ± 0.1%, 3.1 ± 0.1%, and 4.3 ± 0.1%, respectively; *P* = 0.001; Table 5).

We found an increasing trend in the weighted and unadjusted prevalence of metabolic syndrome and high-risk FRS in males and females according to deciles of albuminuria (Fig. 3). The prevalence of metabolic syndrome increased significantly between the seventh (3.89–5.15 mg/g Cr) and eighth (5.16–7.36 mg/g Cr) deciles (*P* = 0.004). The prevalence of high-risk FRS increased significantly between the eighth (5.16–7.36 mg/g Cr) and ninth (7.37–12.09 mg/g Cr) deciles of albuminuria (*P* < 0.001).

## Discussion

We found a significant association between low-grade albuminuria and cardiovascular risk in a nationally representative sample from the 2011–2012 KNHANES. The percentages of hypertension, diabetes, metabolic syndrome, and high-risk FRS increased according to tertile of UACR in individuals with albuminuria below the conventional threshold for microalbuminuria. This positive relationship persisted after excluding participants with hypertension and diabetes. The weighted comparisons among the deciles of UACR revealed that the prevalences of metabolic syndrome and high-risk FRS began to increase at approximately 3.89–5.15 and 5.16–7.36 mg/g Cr, respectively.

Although several prospective studies have shown that low-grade albuminuria below the conventional threshold for microalbuminuria (30 mg/g Cr) is associated with increased cardiovascular risk and mortality in Western populations, few such studies have been conducted in Asian populations [4–6,19]. One previous study showed that low-grade albuminuria was significantly associated with the increasing prevalence of metabolic syndrome and its components in middle-aged and elderly Chinese individuals.[8] Furthermore, low-grade albuminuria has been found to be associated with carotid intima-media thickness (IMT) in Chinese patients with type 2 diabetes [9], and a recent study revealed an association between low-grade albuminuria and carotid IMT in normotensive, euglycemic middle-aged and elderly Chinese subjects [10]. A study of Korean individuals who visited a health promotion center found an association between low-grade albuminuria and a variety of cardiovascular risk factors [11]. The authors reported a positive association between increasing UACR and increasing waist circumference, BMI, and higher values of serum TG, glucose, high sensitivity C-reactive protein, and systolic blood pressure in healthy Korean males with albuminuria <17 μg/mg. Lee *et al*. [20] found that common carotid artery IMT and diameter and brachial-ankle pulse wave velocity were significantly greater in individuals with high normoalbuminuria (UACR 15.0–29.9 mg/g) than in those with normoalbuminuria (UACR <150.0 mg/g).

We confirmed the association between low-grade albuminuria and metabolic syndrome and cardiovascular risk factors in a nationally representative sample of the Korean population. Furthermore, we estimated CHD risk using the FRS, a well-known measure of an individual’s risk for developing cardiovascular disease. An FRS ≥ 20% is considered to be a CHD risk equivalent [18,21]. Thus our findings support previous studies conducted in Asian populations showing an association between low-grade albuminuria and cardiovascular risk factors and/or related components.

The precise pathophysiological mechanisms linking low-grade albuminuria and cardiovascular risk factors are not known. A previous study suggested that albuminuria results from generalized vascular leakiness rather than a specific renal lesion [22]. The passage of albumin and other macromolecules into the vessel wall causes inflammation, lipid accumulation, and eventual atherosclerosis. Furthermore, microalbuminuria has been associated with insulin resistance, aside from its association with arterial hypertension [23]. Insulin resistance is associated with endothelial dysfunction, which may decrease the bioavailability of nitric oxide and impair endothelium-dependent vasodilation [24]. These effects of albuminuria may increase arterial blood pressure and accelerate micro- and macrovascular disease.

Although previous studies have investigated the association between micro- or macroalbuminuria and the risk for cardiovascular disease, those findings may not explain the relationship between lower levels of albuminuria and cardiovascular disease risk. Albuminuria affects the risk for cardiovascular disease on a continuum from the normal range to microalbuminuria. Ruggenenti *et al*. suggested replacing the terms microalbuminuria and macroalbuminuria with the concept of albuminuria-associated disease [23]. Further studies are needed to clarify the association between low-grade albuminuria and cardiovascular risk beyond the definition of “normal range of albuminuria.”

Several previous investigators have suggested revising the definition of microalbuminuria according to the level that increases the risk for cardiovascular disease and death in the general population. The Heart Outcomes Prevention Evaluation (HOPE) study found that an UACR of 4.42 mg/g affected risk for cardiovascular disease [1]. Klausen *et al*. reported that cardiovascular risk increased in participants with a UACR greater than 6 mg/g in the Copenhagen Heart Study [25]. Romundstad *et al*. found that the lowest UACR level significantly associated with all-cause mortality was in the 60th percentile (6.7 μg/mg) [26]. Although the present study was not a prospective observational one, the prevalence of high-risk FRS increased significantly at the UACR of 5.16–7.36 mg/g Cr. At present, it is not clear whether individuals with/without diabetes who have low-grade microalbuminuria should be treated to reduce the degree of albuminuria below the above-mentioned levels. Additional intervention trials are necessary to investigate whether treating low grade albuminuria with angiotensin receptor blocker (ARB) or/and angiotensin converting enzyme (ACE) inhibitor therapy can reduce the risk for cardiovascular disease and mortality.

The major strength of our study was the large nationally representative sample of adult Koreans without micro- or macroalbuminuria. To the best of our knowledge, this is the first nationwide study to show a significant association between low-grade albuminuria and cardiovascular risk using the FRS in an Asian population. Nevertheless, our study had several limitations. First, we could not take into account the use of ARB or/and ACE inhibitors, which may reduce the degree of albuminuria. Second, we used a single urine spot sample to assess the UACR. Third, because this was not a prospective observational study, we could not evaluate the actual incidence of cardiovascular events in individuals without micro- or macroalbuminuria. Fourth, we could not draw an inference of causality between low-grade albuminuria and estimated cardiovascular risk. High risk FRS might be the cause of urinary albumin leakage, leading to low-grade albuminuria, vice versa.

In conclusion, we observed low-grade albuminuria was significantly associated with estimated cardiovascular risk and MS in a nationally representative sample of Koreans. Our results suggest that low-grade albuminuria may be a possible important indicator of cardiovascular risk.
